# Pre-Exercise Carbohydrate or Protein Ingestion Influences Substrate Oxidation but Not Performance or Hunger Compared with Cycling in the Fasted State

**DOI:** 10.3390/nu13041291

**Published:** 2021-04-14

**Authors:** Jeffrey A. Rothschild, Andrew E. Kilding, Sophie C. Broome, Tom Stewart, John B. Cronin, Daniel J. Plews

**Affiliations:** 1 Sports Performance Research Institute New Zealand (SPRINZ), Auckland University of Technology, Auckland 0632, New Zealand; andrew.kilding@aut.ac.nz (A.E.K.); tom.stewart@aut.ac.nz (T.S.); john.cronin@aut.ac.nz (J.B.C.); daniel.plews@aut.ac.nz (D.J.P.); 2 Discipline of Nutrition, Faculty of Medical and Health Sciences, University of Auckland, Auckland 1023, New Zealand; s.broome@auckland.ac.nz; 3 Human Potential Centre, School of Sport and Recreation, Auckland University of Technology, Auckland 1010, New Zealand

**Keywords:** nutrition, exercise, fat oxidation, oxidative stress, isoprostanes

## Abstract

Nutritional intake can influence exercise metabolism and performance, but there is a lack of research comparing protein-rich pre-exercise meals with endurance exercise performed both in the fasted state and following a carbohydrate-rich breakfast. The purpose of this study was to determine the effects of three pre-exercise nutrition strategies on metabolism and exercise capacity during cycling. On three occasions, seventeen trained male cyclists (VO_2peak_ 62.2 ± 5.8 mL·kg^−1^·min^−1^, 31.2 ± 12.4 years, 74.8 ± 9.6 kg) performed twenty minutes of submaximal cycling (4 × 5 min stages at 60%, 80%, and 100% of ventilatory threshold (VT), and 20% of the difference between power at the VT and peak power), followed by 3 × 3 min intervals at 80% peak aerobic power and 3 × 3 min intervals at maximal effort, 30 min after consuming a carbohydrate-rich meal (CARB; 1 g/kg CHO), a protein-rich meal (PROTEIN; 0.45 g/kg protein + 0.24 g/kg fat), or water (FASTED), in a randomized and counter-balanced order. Fat oxidation was lower for CARB compared with FASTED at and below the VT, and compared with PROTEIN at 60% VT. There were no differences between trials for average power during high-intensity intervals (367 ± 51 W, *p* = 0.516). Oxidative stress (F_2_-Isoprostanes), perceived exertion, and hunger were not different between trials. Overall, exercising in the overnight-fasted state increased fat oxidation during submaximal exercise compared with exercise following a CHO-rich breakfast, and pre-exercise protein ingestion allowed similarly high levels of fat oxidation. There were no differences in perceived exertion, hunger, or performance, and we provide novel data showing no influence of pre-exercise nutrition ingestion on exercise-induced oxidative stress.

## 1. Introduction

Nutritional intake before exercise can influence performance and the physiological responses to an exercise session [1]. Exercise performed with reduced carbohydrate (CHO) availability can increase fat oxidation, increase the activation of cell signaling pathways, and promote oxidative adaptations in skeletal muscle [2,3]. At the same time, sufficient CHO ingestion before and/or during exercise is recommended for exercise sessions requiring a high quality, duration, and/or intensity [4]. It is therefore suggested that CHO ingestion be varied according to the goals and type of each exercise session to optimize both training adaptations and acute performance, yet there is wide variance among athletes regarding appropriate nutritional intake before exercise [5].

Strategies to vary CHO availability before exercise include ingesting high- or low-CHO meals, and exercising in the overnight-fasted state. We recently reported nearly two-thirds of endurance athletes (63%) train in the overnight-fasted state, while 72% consume CHO before some or all training sessions, and only 28% ever consume low-CHO meals before exercise [5,6]. Athletes perform fasted-state training primarily to increase fat oxidation and improve gut comfort during exercise, while athletes that avoid fasted training do so because they feel their workout quality deteriorates and/or they will be too hungry during exercise [6]. It is well established that performing low-to-moderate intensity exercise in the overnight-fasted state can induce higher levels of fat oxidation compared with exercise performed following ingestion of CHO [3]. However, several studies have shown fat oxidation during exercise to be similar following protein ingestion compared with a placebo (fasted) condition [7,8]. Therefore, pre-exercise protein ingestion may be an alternative to performing fasted-state training that could reduce hunger while maintaining high levels of fat oxidation. Additional research is needed to better understand differences in substrate oxidation between CHO-fed, protein-fed, and fasted-state training, as previous studies using pre-exercise protein ingestion have either not had a CHO control group [7,8], performed extended exercise at a single intensity [7,8,9], or provided very large (>1000 kcal) pre-exercise meals [10,11].

From a performance standpoint, fed-state exercise generally enhances prolonged (>60 min), but not shorter duration (<60 min) aerobic exercise compared with exercising in the fasted state [12], although few studies have used a high-intensity interval training (HIIT) model to measure performance despite HIIT being performed by virtually all endurance athletes [13]. Total work performed during HIIT has been reported to be increased in the fed, compared with the fasted state during some [14,15] but not all [16] studies. To our knowledge, no studies have compared pre-exercise CHO, protein, and fasted-state training on HIIT work capacity.

More important for athletes and coaches than an acute training session are the longer-term training adaptations. Exercise-induced oxidative stress provides a key signal for the adaptative response to an exercise session with greater exercise-induced oxidative stress being associated with improved adaptations [17,18], but it is unknown how this might be affected by various pre-exercise meals. At rest, a high-CHO meal can evoke a greater postprandial oxidative stress response compared with a high-fat meal [19], while whey protein can enhance endogenous antioxidant enzyme activity [20]. Furthermore, CHO ingestion before and during exercise can decrease exercise-induced oxidative stress during longer-duration moderate-intensity cycling [21]. Therefore, an understanding of how nutrition influences exercise-induced oxidative stress would be valuable as it could inform pre-exercise nutrition strategies.

Research comparing pre-exercise protein with pre-exercise CHO ingestion and fasted-state training across a range of exercise intensities is needed and could help endurance athletes and coaches make better pre-exercise nutrition choices. Accordingly, the aim of this crossover study was to determine the effects of three different pre-exercise nutrition strategies on substrate oxidation, performance during HIIT, and exercise-induced oxidative stress. We hypothesized that the fasted and protein conditions would have the highest fat oxidation, while cycling power during HIIT would be highest following CHO ingestion. Secondary outcomes were to determine the influence of the pre-exercise meal on cycling gross efficiency, heart rate (HR), and rating of perceived exertion (RPE) during moderate and high intensity cycling, along with hunger and gut comfort before and after exercise.

## 2. Materials and Methods

Participants: Seventeen trained cyclists and triathletes participated in this study (31.2 ± 12.4 years, 181.9 ± 6.4 cm, 74.8 ± 9.6 kg, VO_2peak_ 62.2 ± 5.8 mL·kg^−1^·min^−1^, peak aerobic power 425 ± 55 W/5.7 ± 0.6 W·kg^−1^, average weekly training volume 13.6 ± 3.1 h). Participants were required to be 18–55 years old, with a training history of at least 8 h per week for the previous two years, and a VO_2peak_ > 55 mL/kg/min. One participant completed only two of the three trials due to an injury unrelated to this study. Using the PASS 15 software and a 3x3 cross-over design, we estimated that 16 subjects would allow us to detect a difference in fat oxidation of 0.21 g·min^−1^ among the three within-subject conditions, with 90% power and a type I error rate of 0.05. This is based on an F Test, a between-subject standard deviation of σ = 0.2, and a conservative autocorrelation among the repeated measurements of 0.2. We based our assumed means across the three conditions (carbohydrate = 0.34, protein = 0.55, fasted = 0.55) on previous work [22,23]. The standard deviation of the hypothesized means is σ_m_ = 0.099, equating to a Cohen’s ƒ effect size of 0.495. Using the same parameters, we estimated that 12 subjects were required to detect a difference in respiratory exchange ratio (RER), using means (0.91, 0.86, 0.86) and a between-subject standard deviation of σ = 0.04 (σ_m_ = 0.0236; Cohen’s ƒ = 0.59) also estimated from previous work [22,23]. All study protocols and materials were approved by the Auckland University of Technology Ethics Committee (19/420).

Participants reported to the laboratory on four occasions, seven days apart. Participants were asked to refrain from exercise, caffeine, and alcohol 24 h before each visit and kept a 24 h food log in order to replicate dietary intake prior to each testing day. Instructions on keeping a food log were provided.

Visit 1: After obtaining written informed consent and completing a health screening, a graded exercise test was performed to determine maximal oxygen consumption (VO_2peak_). Participants cycled on an electronically braked cycle ergometer (Excalibur Sport, Lode BV, Groningen, The Netherlands) at 60 W for three minutes followed by a 30 W per minute increase until volitional fatigue. Expired gas was collected and analyzed continuously using a computerized metabolic system with mixing chamber (TrueOne2400, ParvoMedics, Sandy, UT, USA), with the VO_2peak_ recorded as the highest 15-s average. Peak power (W_max_) was determined by the workload in the last completed stage plus the workload relative to the time spent in the last incomplete stage [power of completed stage + (30*(seconds at uncompleted stage/60)]. The ventilatory threshold (VT) was identified as the work rate where the ventilatory equivalent for oxygen (V˙E.V˙O_2_^−1^) began to increase in the absence of changes in the ventilatory equivalent for carbon dioxide (V˙E.V˙CO_2_^−1^), with 15 W deducted to account for the lag in VO_2_ during the incremental test [24].

Following a 10-min rest, participants were familiarized with the HIIT protocol using 3 × 3-min intervals. The first interval was set at 80% W_max_, performed in a cadence-independent manner, while subsequent intervals used the cadence-dependent linear mode set to produce a workload of 80% W_max_ at their preferred cadence. Three intervals were deemed appropriate to minimize the likelihood of training effects and because the participants were fatigued from the VO_2peak_ testing. Participants were asked at the start of the session about their weekly training volume, recorded as self-reported hours per week, and how often they perform exercise in the overnight-fasted state (i.e., without ingesting any calorie-containing foods or beverages). All trials were conducted under standard laboratory conditions (17–19 °C, 40–65% relative humidity), with participants fan cooled during exercise.

Visits 2–4: Participants reported to the laboratory in an overnight-fasted state (~10 h), with each visit at the same time of day. Upon arrival, participants completed a five-question survey that assessed fatigue, sleep quality, muscle soreness, stress, and mood on a five-point scale (scores 1 to 5), with overall well-being determined by summing the five scores [25]. Participants also rated their subjective sensations of hunger and gut discomfort upon arrival and again at the end of each session using paper-based visual analogue scales (VAS) with written anchors of “not hungry at all”/“no discomfort” and “extremely hungry”/“extreme discomfort” placed 0 and 100 mm, respectively [26]. A urine sample was obtained upon arrival (before meal consumption) and within five minutes of completing the exercise session.

In a randomized and counter-balanced order, participants received one of three meals to be consumed within a 5-min window. A CHO-rich meal (CARB; 1 g/kg CHO), a protein-rich meal (PROTEIN; 0.45 g/kg protein + 0.24 g/kg fat), or 500 mL water (FASTED). A 70-kg person received 51 g white bread (Tip Top, New Zealand) with 19 g raspberry jam (Barkers, New Zealand) and 500 mL of a 7% CHO-electrolyte drink [4:1 glucose-to-fructose ratio; Replace, Horleys, New Zealand] for CARB, and 25 g whey protein isolate (ICE, Horleys, New Zealand) with 33 g peanut butter (Forty Thieves, New Zealand) and 500 mL water for PROTEIN. A small amount of fat was included with PROTEIN to keep the two trials isocaloric and mimic real-world application. Total energy content of the CARB and PROTEIN meals was 4 kcal per kg body mass (299 ± 38 kcal). All groups consumed 500 mL fluid and could drink water ad libitum during the remainder of the session.

**Figure 1:**
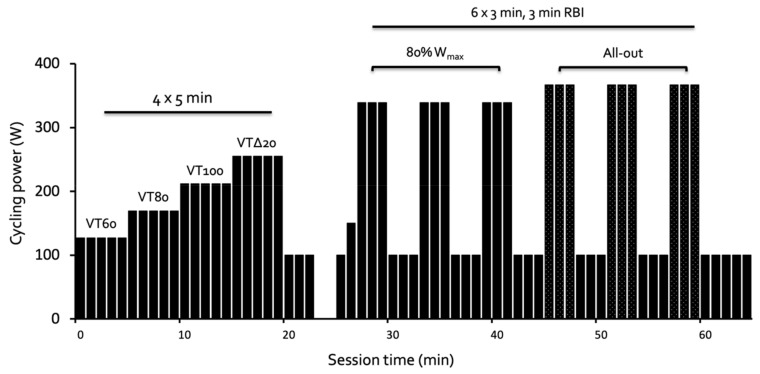
Schematic overview of cycle testing sessions using wattage from an example participant. The 5-min intervals were performed at intensities equivalent to 60%, 80%, and 100% of their ventilatory threshold (VT 60, VT80, VT100, respectively), and 20% of the difference between the ventilatory threshold and peak power (W_max_, VTΔ20), followed by a 3-min cool down at 100 W. The 3-min intervals included a lead-in of 1 min at 100 W and 1 min at 150 W, followed by intervals 1–3 at 80% of Wmax, and intervals 4–6 performed as maximal efforts.

Thirty minutes after ingestion of the meal, participants began the sub-maximal cycling portion of the testing which included 4 × 5-min stages at a power equivalent to 60%, 80%, and 100% of VT (VT60, VT80, VT100, respectively), and 20% of the difference between VT and W_max_ (VTΔ20), to measure substrate oxidation, energy expenditure, heart rate (HR), and perceived exertion (RPE) (Figure 1). Expired gas was continuously measured using a metabolic cart (TrueOne2400, ParvoMedics, Sandy, UT, USA), with average values during the final two minutes of each stage analyzed. Intensity was normalized to the VT to reduce inter-subject variability in the physiological and perceived responses to exercise compared with using a percentage of VO_2peak_ [27].

Rate of energy expenditure (EE) was calculated using the formulas of Jeukendrup and Wallis [28], with cycling gross efficiency (GE) calculated as GE(%) = (mechanical work (kcal/min)/energy expenditure (kcal/min)) × 100. Whole-body rates of CHO and fat oxidation were calculated using standard equations, assuming 9.75 kcal/g fat and 4.07 kcal/g CHO [28].

Following a 3-min static rest, participants performed 6 × 3-min cycling intervals with 3 min of active recovery (100 W) between each interval (Figure 1). The first three intervals were performed at 80% of W_max_, in a cadence-independent manner. Intervals 4–6 used the cadence-dependent linear mode set to produce a workload of 80% W_max_ at their preferred cadence, with participants instructed to produce their maximal power output across intervals 4–6 by increasing the cycling cadence. Immediately following intervals 3 and 6, a 0.3μL blood sample was collected from the left index fingertip and analyzed immediately using a portable blood lactate analyzer (Lactate Pro 2, Carlton, Australia). Power output (W) during HIIT was analyzed as mean power (W) for each interval. Heart rate was measured using a chest-strap (Polar T31, Polar, Inc., Kempele, Finland), with average values during the final 30 s of each interval analyzed. Rating of perceived exertion (RPE) was recorded following each submaximal stage and each high-intensity interval using Borg’s 6–20 scale [29], and at the end of the session (_S_RPE) using a 10-point scale [30]. We chose to have the work rate “clamped” for the first three intervals to compare HR, RPE, and lactate across conditions at a fixed cycling power, and have three intervals performed as maximal efforts to be used to determine work capacity during HIIT.

A competitive immunoassay was used for the quantitation of urinary F_2_-isoprostanes (Kit #51635, Cayman Chemicals, Ann Arbor, MI, USA) as previously described [31]. Samples were purified using solid-phase extraction cartridges. For standardizing urine dilution, creatinine levels were measured using a commercially available kit (Kit #500701, Cayman Chemicals, Ann Arbor, MI, USA). Due to technical problems the number of samples analyzed was n = 12 for CARB, n = 12 for FASTED, and n = 11 for PROTEIN.

Statistical analysis: A series of linear mixed models were used to estimate differences in the exercise-induced changes between the three treatment conditions (CARB, PROTEIN, FASTED). These were fit using the lme4 R package. For the submaximal portion, intensity (four levels: VT60, VT80, VT100, VTΔ20) was added as a fixed effect, while interval (three levels) was considered a fixed effect for the high-intensity portion. When examining differences pre-post exercise (for hunger, gut comfort, and oxidative stress measures), time point (two levels; pre and post) was added as a fixed effect. For all models, treatment order was included as a fixed effect (given the crossover design) and participant ID was specified as a random intercept. Interactions between the treatment and other fixed effects were explored, and the optimal, best-fitting model for each outcome was decided based on the likelihood ratio test. The fit of each model was checked by visualizing the Q–Q and other residual plots to ensure approximate residual normality and heteroscedasticity, using the performance R package. Model-estimated means were calculated using the emmeans R package and presented as estimated means ± 95% confidence interval (CI). Contrasts between each treatment (within each intensity, interval, or time point) were estimated, with multiple comparisons adjusted using the Holm correction. A standardized effect size for each contrast (delta total variance; 𝛿t) was computed by dividing the mean difference by the population SD (calculated as the square root of the sum of the variance components of the random effects) [32]. Effect size are interpreted as small (0.2), medium (0.5) and large (0.8) [33]. One participant was excluded from the submaximal mixed models because their intensity was more than 3 SDs above the mean, due to an overestimation of the VT. The level of significance for all analyses was set at *p* < 0.05, and all analyses were carried out with R version 4.0.3.

## 3. Results

### 3.1. Submaximal Exercise

**Figure 2:**
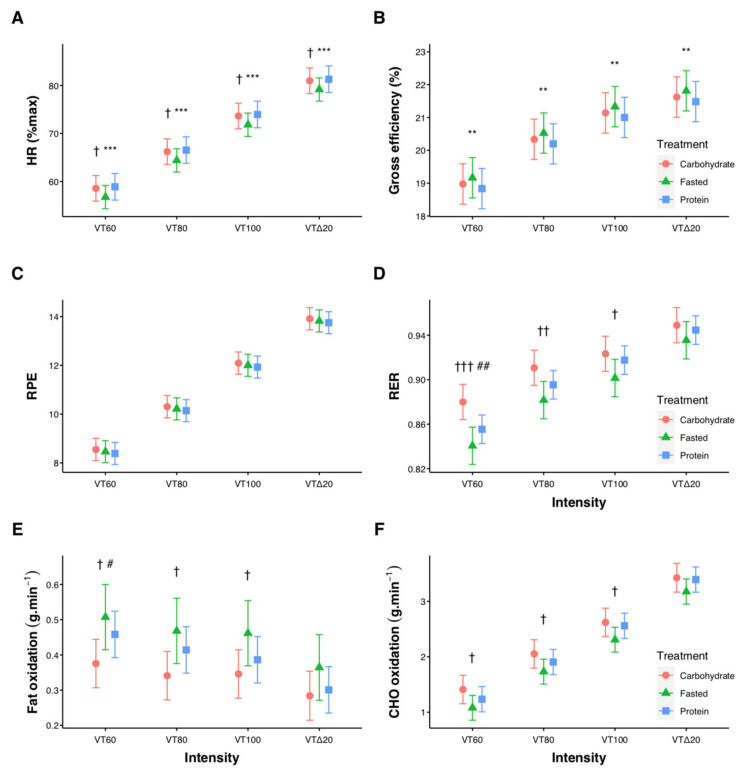
Influence of pre-exercise nutrition interventions on heart rate (HR, (**A**)), cycling gross efficiency (**B**), rating of perceived exertion (RPE, (**C**)), respiratory exchange ratio (RER, (**D**)), fat oxidation (**E**), and carbohydrate (CHO) oxidation (**F**) during submaximal cycling. Significant differences CARB vs. FASTED († *p* < 0.05, †† *p* < 0.01, ††† *p* < 0.001), significant differences CARB vs. PROTEIN (# *p* < 0.05, ## *p* < 0.01), significant differences FASTED vs. PROTEIN (** *p* < 0.01, *** *p* < 0.001). Error bars represent 95% confidence intervals of the model-estimated mean.

Relative exercise intensity for the four submaximal stages was 40.8 ± 4.4, 50.6 ± 5.9, 60.6 ± 7.2, and 71.8 ± 6.3%VO_2peak_. For HR, RPE, RER, energy expenditure, VO_2_, and CHO oxidation there were significant pairwise differences between each intensity level (all *p* < 0.001, Figure 2). Contrasts between treatments at each intensity revealed HR, measured as percentage of each individual’s maximal HR, was lower for FASTED compared with both CARB and PROTEIN (*p* < 0.05, Figure 2A). Gross cycling efficiency was higher (*p* < 0.01) for FASTED compared with PROTEIN at each intensity (Figure 2B), while RPE was not different between treatments (Figure 2C). RER was higher (*p* < 0.05) for CARB compared with FASTED at VT60, VT80, and VT100, and compared with PROTEIN at VT60 (Figure 2D). Fat oxidation was lower (*p* < 0.05) for CARB compared with FASTED at VT60, VT80, and VT100, and compared with PROTEIN at VT60 (Figure 2E). Carbohydrate oxidation was different (*p* < 0.05) between CARB and FASTED at VT60, VT80, and VT100 (Figure 2F). Table 1 shows effect sizes with 95% confidence intervals for all contrasts at each submaximal intensity. Estimated means, confidence intervals, and *p*-values for all submaximal values are provided in Supplementary Materials Tables S1–S7.

**Figure 3:**
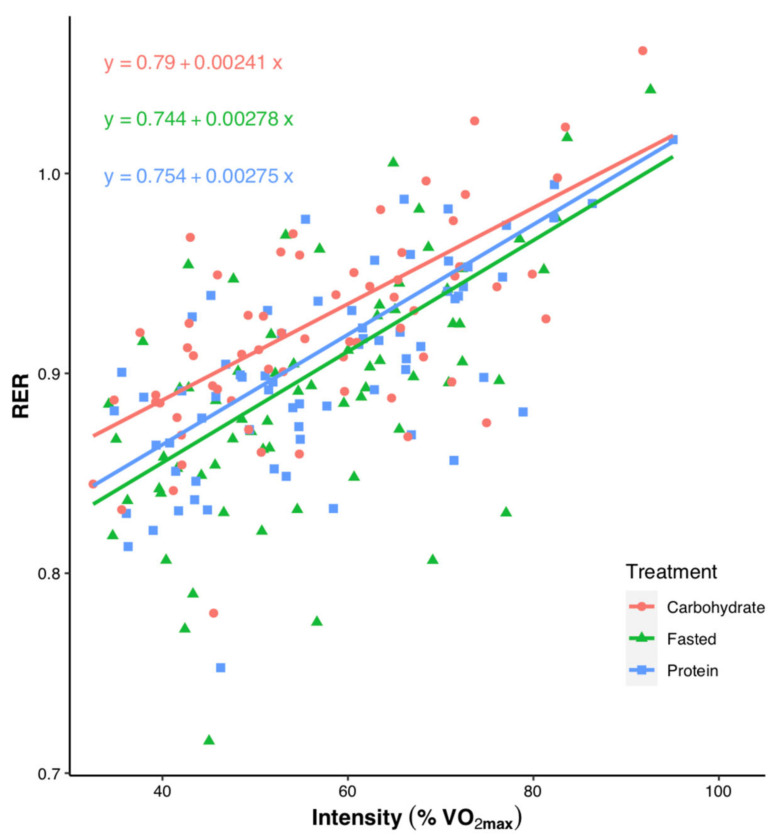
Individual data points showing correlations between each individual’s relative exercise intensity as a percentage of VO_2peak_ and respiratory exchange ratio (RER) during submaximal exercise, separated by treatment. Significant effects are found for treatment (*p* < 0.001) and intensity (*p* < 0.001). Trend line is based on model-estimated values.

Individual data points for RER across the submaximal exercise stages in relation to each individual’s VO_2peak_ are shown in Figure 3. There was a significant effect of both treatment and intensity on RER (*p* < 0.001), but no significant interaction between treatment and intensity (*p* = 0.283).

### 3.2. High-Intensity Exercise

**Figure 4:**
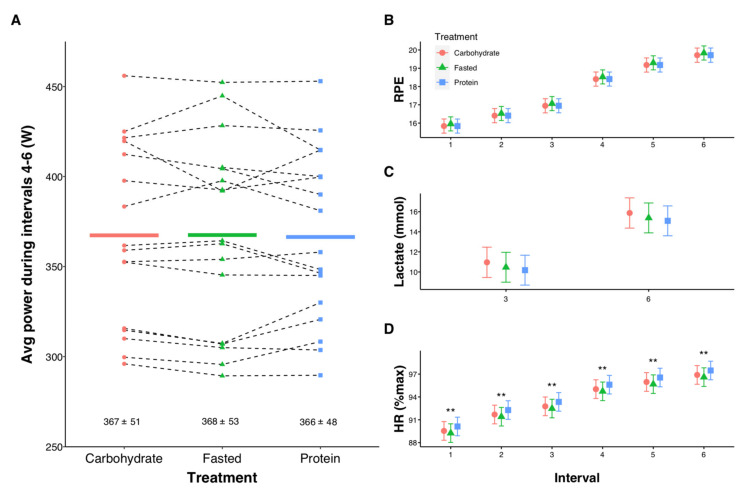
Influence of pre-exercise nutrition interventions on mean power during three all-out 3-min efforts (**A**), rating of perceived exertion (RPE, **B**), lactate (**C**), and heart rate (HR, **D**) during high-intensity interval training. In (**A**) individual data points are shown with solid lines representing means and numeric values reflecting mean ± SD. In (**B**–**D**), error bars represent 95% confidence intervals of the model-estimated mean. ** Significant differences for FASTED vs. PROTEIN (*p* < 0.01).

Intervals 1–3 were performed at 80%W_max_, corresponding to 340 ± 44 W. The subsequent three intervals were performed as maximal efforts, with no differences between treatments for average power (*p* = 0.516, Figure 4A). Similarly, there were no differences in RPE or lactate (Figure 4B,C) between trials. However, contrasts between treatments showed HR was higher during PROTEIN compared with FASTED at each interval (all *p* = 0.004, Figure 4D).

### 3.3. Pre-Post Exercise

**Figure 5:**
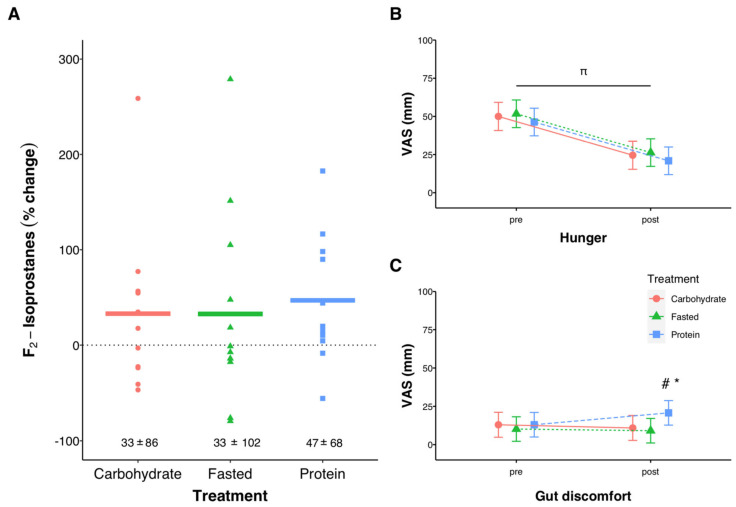
Urinary F_2_-Isprostane percent change (pre to post-exercise, (**A**)), and subjective levels of hunger (**B**) and gut discomfort (**C**) before and after exercise using a visual analog scale (VAS). All measurements at ‘pre’ were taken upon arrival to the lab in the overnight-fasted state, before being provided any nutrition treatment. In (**A**) individual data points are shown with solid lines representing means and the numeric values reflecting mean ± SD. In (**B**,**C**) error bars represent 95% confidence intervals of the model-estimated mean. ^π^ significant effect of time (*p* < 0.001), significant effects of treatment (^#^ PROTEIN vs. CARB, *p* = 0.012, * PROTEIN vs. FASTED, *p* = 0.032) at the post time point. For (**A**), *n* = 12 for Carbohydrate, *n* = 12 for Fasted, and *n* = 11 for Protein.

There was no effect of exercise (*p* = 0.510) or treatment (*p* = 0.595) on urinary F_2_-Isoprostanes (Figure 5A). Hunger decreased from pre to post exercise (*p* < 0.001), with no effect of treatment, while gut discomfort following exercise was higher with protein compared with FASTED (*p* = 0.032) and CARB (*p* = 0.012, Figure 5B,C). Overall session RPE was not different between trials (*p* = 0.076), but there was a trend for CARB (7.9 (95%CI 7.5, 8.2) to be lower than FASTED (8.3 (95% CI 7.9, 8.5), *p* = 0.101, data not shown). When asked how often they typically perform exercise in the overnight-fasted state 59% of participants reported “rarely or never (less than 1x per week)”, 18% reported “sometimes (1–2x per week)”, and 24% reported “often or always (>2x per week)”.

## 4. Discussion

To our knowledge, this is the first study to compare continuous exercise and HIIT performed in the overnight-fasted state with both CHO-rich and protein-rich pre-exercise meals. These data reveal exercising in the overnight-fasted state can increase fat oxidation during submaximal exercise compared with exercising following a CHO-rich breakfast, and that pre-exercise protein ingestion allows similarly high levels of fat oxidation. Furthermore, there were no between-group differences in RPE, hunger, or performance during HIIT, and we provide novel data showing no influence of pre-exercise nutrition ingestion on F_2_-Isoprostanes, a measure of exercise-induced oxidative stress.

It is well established that exercising in the fasted state allows higher levels of fat oxidation than exercise performed in the CHO-fed state during low-to-moderate intensity exercise, with these differences reduced as exercise intensity increases [1,3]. Accordingly, we found the FASTED group had a lower RER (and thus higher levels of fat oxidation) compared with CARB at and below the VT, but not above (Figure 2D). Ingestion of PROTEIN resulted in a lower RER than CARB at the lowest intensity, but differences between groups were reduced as the intensity increased. Fat oxidation for PROTEIN was numerically lower, but not significantly different than FASTED (e.g., 0.46 vs. 0.51 g.min^−1^ at VT60). The lack of statistical significance is likely due to the wide variation in RER observed between participants (Figure 3). In line with our findings, others have reported a protein-rich pre-exercise meal increased fat oxidation during moderate-intensity exercise compared with a CHO-rich meal [34], and there were no differences in fat oxidation when consuming whey protein before and during steady-state cycling compared with a placebo (fasted) trial [7]. Similar levels of fat oxidation between fasted and protein-fed exercise have also been reported during cycling between 58%–86% VO_2max_ [10], running at 55%–60% heart rate reserve [9], and cycling at ~50% VO_2max_ in a glycogen-depleted state [8]. A primary reason endurance athletes perform fasted-state training is a desire to increase fat oxidation during exercise [6], and our findings provide further evidence supporting the use of both pre-exercise protein ingestion and fasted-state training to increase fat oxidation during low-intensity exercise compared with a CHO-rich breakfast, which likely impairs fat oxidation.

Decreased cycling efficiency was observed in PROTEIN compared with FASTED, however the practical relevance of these differences is likely minimal. This may be accounted for by meal-induced thermogenesis, which is increased during exercise [35] and is nearly three-fold higher following protein compared with CHO ingestion [36,37]. We also observed elevated HR in both fed conditions (Figure 2A), which could be related to the increased meal-induced thermogenesis, and/or increased sympathetic nervous system activity following glucose ingestion [38].

It is more common for athletes to perform lower-intensity exercise in the overnight-fasted state, as many feel their performance during higher-intensity exercise will be diminished [6]. Despite this belief, we found work capacity and RPE during HIIT were not different between trials. It is possible the assumption of diminished work capacity during fasted-state HIIT comes from observations of decreased power during HIIT performed with reduced muscle glycogen concentrations [39,40]. However, exercise performed in the overnight-fasted state lowers hepatic but not muscle glycogen [41], and therefore fasted exercise with normal muscle glycogen levels would not be expected to have the performance decrement. Similar to our findings, no effect of a mixed macronutrient breakfast on HIIT performance or RPE has been reported compared with exercising in the overnight-fasted state [16]. However, others have shown benefit of pre-exercise CHO ingestion on an exercise capacity test lasting ~8–10 min [42]. Factors that influence central fatigue may be important during short-duration exercise, which is not limited by glycogen depletion. Central fatigue may be reduced by ingesting CHO [43] or branched-chain amino acids [44]. In our study work capacity was not influenced by pre-exercise CHO or protein ingestion, but it is possible that longer-duration exercise would have revealed differences between treatments.

Many athletes perform fasted-state training to improve gut comfort during exercise, while one of the most common reasons for avoiding training in the fasted state is related to hunger [6]. To this end, we obtained subjective ratings of hunger and gut comfort before and after exercise using a VAS. Hunger decreased following exercise, with no differences between trials. It is possible that post-exercise hunger scores would have been higher following lower-intensity exercise, as blood lactate accumulation is associated with the suppression of the hunger hormone ghrelin and subjective appetite [45]. Indeed, sprint interval exercise has induced a greater suppression of appetite during exercise compared with continuous endurance exercise [46]. Following moderate-intensity exercise there has been reduced hunger with a CHO-rich pre-exercise meal compared with fasted-state exercise [47], and with a protein-rich compared with CHO-rich pre-exercise meal [34]. Gut discomfort in our study was low before exercise (~12 out of 100) and increased following exercise only in PROTEIN. Protein is known to increase the risk of gut discomfort during exercise in some [48,49], but not all [7], studies. However, in our study, the observed increase was modest (post-exercise gut discomfort ~21 out of 100), suggesting pre-exercise protein ingestion could still be an effective strategy for those who would prefer to eat before exercise while maintaining higher levels of fat oxidation.

Despite the increase in fat oxidation and maintenance of HIIT performance, exercising in the overnight-fasted state could more likely lead to a negative energy balance, which can be associated with hormonal and immune dysfunction [50]. For example, the average athlete in this study riding at the first ventilatory threshold will burn ~850 kcal per hour, creating a large calorie deficit when exercising in the fasted state. Even for the athlete who can achieve energy balance, body composition may be influenced by large or frequent within-day energy deficits [51]. The results of this study therefore highlight the utility of pre-exercise protein ingestion as a method of providing energy intake while still allowing higher levels of fat oxidation, particularly for those who want to increase fat burning without incurring a large caloric deficit.

Reactive oxygen species play a direct role in regulating the response to acute exercise and are critical for longer-term exercise training adaptations [18,52,53]. We found no effect of nutrition or exercise on F_2_-Isoprostanes, one of the preferred markers for the detection of organism-wide oxidative stress [54]. Similar to others [55], we observed a large degree of inter-individual variability in exercise-induced oxidative stress (Figure 5A). It is potentially surprising that we didn’t see an increase in F_2_-Isoprostanes following exercise as most, but not all, studies have reported exercise-induced increases [54]. This result could be due to the well-trained status of the participants [56], the exercise protocol (HIIT lasting < 60 min), or the sample size and high interindividual variability in the response. It is important to further investigate the effects of pre-exercise nutrition on exercise-induced oxidative stress, potentially using different oxidative stress markers or different exercise protocols (e.g., sprint interval training, and/or longer-duration continuous exercise), as this could have implications for longer-term training adaptations.

The size and timing of the nutrient ingestion was chosen to maximize ecological validity. Ingesting a small amount of CHO (e.g., 1 g/kg) just prior to exercise (e.g., 30 min) is similar to the day-to-day practices of endurance athletes [5] and in contrast with previous studies using extremely large test meals [11] provided up to 4 h prior to exercise [57]. No differences in performance have been observed when CHO was consumed 15, 45, or 75 min [58], 15 or 60 min [59], or 5 or 35 min [60] before exercise. There also appears to be no effect of meal size on substrate oxidation during exercise, as similar values were found with 45 and 156 g of CHO consumed 4 h prior to exercise [61], and 25, 75, or 200 g of CHO consumed 45 min prior to exercise [62]. Therefore, our findings should be generalizable to a range of pre-exercise meal sizes and timings.

The potential for placebo effects must be acknowledged, as it is challenging to blind participants to their treatments when using solid and/or familiar foods (e.g., jam sandwich or peanut butter). Cycling time-trial performance has improved when subjects perceived they had consumed breakfast (either a viscous placebo or 2 g/kg CHO) before exercise, compared with a water-only trial [63]. A placebo effect is more likely during short-duration exercise when muscle glycogen use is not a limiting factor for performance [63,64,65]. The duration of exercise in our study was 60 min, with roughly 30% of that time spent at high intensity implying glycogen depletion was not a limiting factor for performance. Although only seven out of 17 study participants reported regularly performing training sessions in the overnight-fasted state, there were no performance differences when accounting for their habitual use of fasted training (data not shown). In a survey of endurance athletes, 26% agreed and 51% disagreed with the statement, “the quality of my workout is the same whether I eat or do not eat beforehand” [6]. Therefore, it is likely a large inter-individual variation exists with regard to the perception of breakfast and its influence on performance. However, is must also be acknowledged that the influence of the pre-exercise meal on performance will be related to duration as well as intensity of exercise [1,12].

Future studies are needed to extend these findings to other populations including women and untrained individuals. There are known sex differences in substrate use and molecular signaling during exercise [66,67], and trained athletes have a greater capacity for fat oxidation compared with untrained or recreationally active populations [68]. Additionally, calculations of energy expenditure assume negligible contribution of protein oxidation [28]. Although not quantified in this study, there is the possibility of increased protein oxidation during exercise in the PROTEIN group that may have been unaccounted for. This protein oxidation may to some extent account for the reduction in GE in the PROTEIN group. It has been reported that protein oxidation contributes up to 10% of total oxygen consumption [69], and can vary depending on training status [70], habitual diet [71], muscle glycogen levels [72], and pre-exercise protein ingestion [73], but further quantification of the influence of the pre-exercise meal is needed. Finally, training studies are needed to determine if longer-term adaptations to continuous and/or HIIT may be differentially influenced by pre-exercise nutrition choices.

## 5. Conclusions

In summary, fat oxidation during submaximal exercise was highest in the overnight-fasted state and following pre-exercise protein ingestion. There were no differences in work capacity, RPE, oxidative stress, or hunger between treatments. Consuming a low-CHO meal before submaximal exercise will not meaningfully impair fat oxidation and eating a high- or low-CHO meal does not confer additional performance benefit during HIIT compared with training in the overnight-fasted state. Therefore, athletes who wish to increase fat oxidation but promote energy balance can use pre-exercise protein ingestion as a viable alternative to fasted training sessions. Conversely, for shorter duration higher intensity sessions, fasted training and protein ingestion are also viable choices as performance was not compromised, suggesting athletes can choose whether to eat based on personal preference. However, longer-term training studies are needed as the net adaptive responses of chronic CHO or protein ingestion prior to exercise is unknown.

## Figures and Tables

**Table 1:** Effect sizes [95% confidence intervals] for all contrasts during submaximal exercise.

Contrast	Intensity	Heart Rate	Gross Efficiency	RPE	RER	Fat Oxidation (g/min)	Carbohydrate Oxidation (g/min)
**CARB-FASTED**	**VT60**	Small	Trivial	Trivial	Large	Large	Medium
		ES = 0.34	ES = −0.15	ES = 0.08	ES = 1.29	ES = −0.85	ES = 0.69
		[0.07, 0.61]	[−0.32, 0.02]	[−0.23, 0.39]	[0.69, 1.89]	[−1.46, −0.25]	[0.18, 1.21]
**CARB-PROTEIN**	**VT60**	Trivial	Trivial	Trivial	Large	Medium	Small
		ES = −0.06	ES = 0.11	ES = 0.15	ES = 0.80	ES = −0.54	ES = 0.37
		[−0.37, 0.25]	[−0.06, 0.28]	[−0.16, 0.45]	[0.28, 1.33]	[−1, −0.07]	[−0.1, 0.84]
**FASTED-PROTEIN**	**VT60**	Small	Small	Trivial	Small	Small	Small
		ES = −0.4	ES = 0.26	ES = 0.07	ES = −0.49	ES = 0.32	ES = −0.33
		[−0.55, −0.24]	[0.09, 0.42]	[−0.23, 0.37]	[−1.03, 0.06]	[−0.2, 0.84]	[−0.82, 0.17]
**CARB-FASTED**	**VT80**	Small	Trivial	Trivial	Large	Large	Medium
		ES = 0.34	ES = −0.15	ES = 0.08	ES = 0.95	ES = −0.82	ES = 0.67
		[0.07, 0.61]	[−0.32, 0.02]	[−0.23, 0.39]	[0.36, 1.54]	[−1.43, −0.22]	[0.16, 1.19]
**CARB-PROTEIN**	**VT80**	Trivial	Trivial	Trivial	Medium	Small	Small
		ES = −0.06	ES = 0.11	ES = 0.15	ES = 0.50	ES = −0.47	ES = 0.31
		[−0.37, 0.25]	[−0.06, 0.28]	[−0.16, 0.45]	[−0.02, 1.02]	[−0.94, −0.01]	[−0.16, 0.77]
**FASTED-PROTEIN**	**VT80**	Small	Small	Trivial	Small	Small	Small
		ES = −0.4	ES = 0.26	ES = 0.07	ES = −0.45	ES = 0.35	ES = −0.37
		[−0.55, −0.24]	[0.09, 0.42]	[−0.23, 0.37]	[−0.99, 0.09]	[−0.17, 0.87]	[−0.86, 0.13]
**CARB-FASTED**	**VT100**	Small	Trivial	Trivial	Medium	Medium	Medium
		ES = 0.34	ES = −0.15	ES = 0.08	ES = 0.71	ES = −0.75	ES = 0.66
		[0.07, 0.61]	[−0.32, 0.02]	[−0.23, 0.39]	[0.13, 1.3]	[−1.35, −0.15]	[0.14, 1.17]
**CARB-PROTEIN**	**VT100**	Trivial	Trivial	Trivial	Trivial	Small	Trivial
		ES = −0.06	ES = 0.11	ES = 0.15	ES = 0.19	ES = −0.26	ES = 0.13
		[−0.37, 0.25]	[−0.06, 0.28]	[−0.16, 0.45]	[−0.33, 0.7]	[−0.73, 0.2]	[−0.34, 0.59]
**FASTED-PROTEIN**	**VT100**	Small	Small	Trivial	Medium	Small	Medium
		ES = −0.4	ES = 0.26	ES = 0.07	ES = −0.53	ES = 0.49	ES = −0.53
		[−0.55, −0.24]	[0.09, 0.42]	[−0.23, 0.37]	[−1.07, 0.01]	[−0.03, 1.01]	[−1.02, −0.03]
**CARB-FASTED**	**VTΔ20**	Small	Trivial	Trivial	Small	Medium	Medium
		ES = 0.34	ES = −0.15	ES = 0.08	ES = 0.44	ES = −0.52	ES = 0.53
		[0.07, 0.61]	[−0.32, 0.02]	[−0.23, 0.39]	[−0.14, 1.03]	[−1.13, 0.09]	[0, 1.05]
**CARB-PROTEIN**	**VTΔ20**	Trivial	Trivial	Trivial	Trivial	Trivial	Trivial
		ES = −0.06	ES = 0.11	ES = 0.15	ES = 0.14	ES = −0.11	ES = 0.07
		[−0.37, 0.25]	[−0.06, 0.28]	[−0.16, 0.45]	[−0.37, 0.66]	[−0.58, 0.36]	[−0.4, 0.54]
**FASTED-PROTEIN**	**VTΔ20**	Small	Small	Trivial	Small	Small	Small
		ES = −0.4	ES = 0.26	ES = 0.07	ES = −0.30	ES = 0.41	ES = −0.46
		[−0.55, −0.24]	[0.09, 0.42]	[−0.23, 0.37]	[−0.84, 0.24]	[−0.11, 0.94]	[−0.96, 0.04]

Submaximal exercise consisted of 4 × 5-min stages at a power equivalent to 60%, 80%, and 100% of the first ventilatory threshold (VT) (VT60, VT80, VT100, respectively), and 20% of the difference between VT and Wmax (VTΔ20). RER: Respiratory exchange ratio, RPE: Rating of perceived exertion.

## Data Availability

Data can be made available on request.

## Supplementary Materials

The following are available online at https://www.mdpi.com/article/10.3390/nu13041291/s1, Table S1: Estimated means and confidence intervals for heart rate during submaximal exercise, Table S2: Estimated means and confidence intervals for gross efficiency during submaximal exercise, Table S3: Estimated means and confidence intervals for rating of perceived exertion during submaximal exercise, Table S4: Estimated means and confidence intervals for respiratory exchange ratio during submaximal exercise, Table S5: Estimated means and confidence intervals for fat oxidation (g/min) during submaximal exercise, Table S6: Estimated means and confidence intervals for carbohydrate oxidation during submaximal exercise, Table S7: Holm-adjusted p-values for all contrasts during submaximal exercise.
